# Beta carbonic anhydrases: novel targets for pesticides and anti-parasitic agents in agriculture and livestock husbandry

**DOI:** 10.1186/1756-3305-7-403

**Published:** 2014-08-29

**Authors:** Reza Zolfaghari Emameh, Harlan Barker, Vesa P Hytönen, Martti E E Tolvanen, Seppo Parkkila

**Affiliations:** School of Medicine, University of Tampere, 33520 Tampere, Finland; BioMediTech, University of Tampere, 33520 Tampere, Finland; Fimlab Laboratories Ltd and Tampere University Hospital, Biokatu 4, 33520 Tampere, Finland; Department of Information Technology, University of Turku, 20014 Turku, Finland

**Keywords:** Beta carbonic anhydrase, Inhibitors, Insecticides, Pesticides, Anti-parasitic agents, Agriculture, Livestock husbandry

## Abstract

**Background:**

The genomes of many insect and parasite species contain beta carbonic anhydrase (β-CA) protein coding sequences. The lack of β-CA proteins in mammals makes them interesting target proteins for inhibition in treatment of some infectious diseases and pests. Many insects and parasites represent important pests for agriculture and cause enormous economic damage worldwide. Meanwhile, pollution of the environment by old pesticides, emergence of strains resistant to them, and their off-target effects are major challenges for agriculture and society.

**Methods:**

In this study, we analyzed a multiple sequence alignment of 31 β-CAs from insects, some parasites, and selected plant species relevant to agriculture and livestock husbandry. Using bioinformatics tools a phylogenetic tree was generated and the subcellular localizations and antigenic sites of each protein were predicted. Structural models for β-CAs of *Ancylostoma caninum*, *Ascaris suum*, *Trichinella spiralis*, and *Entamoeba histolytica*, were built using *Pisum sativum* and *Mycobacterium tuberculosis* β-CAs as templates.

**Results:**

Six β-CAs of insects and parasites and six β-CAs of plants are predicted to be mitochondrial and chloroplastic, respectively, and thus may be involved in important metabolic functions. All 31 sequences showed the presence of the highly conserved β-CA active site sequence motifs, CXDXR and HXXC (C: cysteine, D: aspartic acid, R: arginine, H: histidine, X: any residue). We discovered that these two motifs are more antigenic than others. Homology models suggested that these motifs are mostly buried and thus not well accessible for recognition by antibodies.

**Conclusions:**

The predicted mitochondrial localization of several β-CAs and hidden antigenic epitopes within the protein molecule, suggest that they may not be considered major targets for vaccines. Instead, they are promising candidate enzymes for small-molecule inhibitors which can easily penetrate the cell membrane. Based on current knowledge, we conclude that β-CAs are potential targets for development of small molecule pesticides or anti-parasitic agents with minimal side effects on vertebrates.

## Background

Various pests, including weeds, insects, and plant viruses, often reduce crop production by 25-50% [1, 2]. On the other hand, the widespread use of synthetic insecticides for controlling pests produces many negative consequences (e.g. insecticide resistance, toxicity to mammals and other non-target animals, residue problems, and environmental pollution). High risk groups exposed to pesticides include: production workers, formulators, sprayers, mixers, loaders, and agricultural farm workers. Residual pesticides represent a real threat for human health. When 9700 samples of fruits and vegetables were analyzed for seven pesticides (Acephate, Chlopyriphos, Chlopyriphos-methyl, Methamidophos, Iprodione, Procymidone, and Chlorothalonil), 5.2% of the samples were found to contain residues. Pesticides can contaminate soil, water, and turf. In addition to killing insects or weeds, pesticides can be toxic to other organisms including birds, fish, beneficial insects, and non-target plants [3]. The extensive use of pesticides, such as Dichlorodiphenyltrichloroethane (DDT), in recent decades has led to their recurrent detection in many surface and ground waters [4]. As a result of these negative consequences, natural products have become popular among consumers [5].

As of the 1960s pesticide resistance had already evolved in some key greenhouse pests, prompting the development of alternative methods of management. The pressure to reduce insecticide usage was reinforced by the adoption of bumble-bees for pollination within greenhouses [6]. Biological control plays a central role in the production of many greenhouse crops. The term “Biopesticide” encompasses a broad array of microbial pesticides, including biochemicals derived from micro-organisms and other natural sources, and those resulting from the incorporation of DNA into various agricultural commodities [7]. Bacteria, fungi, viruses, entomopathogenic nematodes (ENPs), and herbal essential oils are often used as bio-pesticides [8]. Novel approaches to control pests involve targeting of specific insect and parasite enzymes. This can be done using either chemical or biological compounds. Acetylcholinesterase (AChE) of the malaria mosquito (*Anopheles gambiae*) has been reported as a target site for pesticides [9]. Three pesticides, Atrazine, DDT, and Chlorpyrifos, have been determined to affect the esterase (GE), glutathione S-transferase (GST), cytochrome P450 monooxygenase (P450), and acetylcholinesterase (AChE) activities of *Chironomus tentans* (an aquatic midge) [4]. Proteinases serving as insect digestive enzymes are defined targets in pest control [10]. Enzyme inhibitors, such as: piperonyl butoxide (PB), a mixed-function oxidase (MFO) inhibitor; triphenyl phosphate (TPP), a carboxyesterase (CarE) inhibitor; and diethyl maleate (DEM), a glutathione S-transferase (GST) inhibitor, have been used to inhibit insect enzymes [11]. Inhibition of *Plasmodium falciparum* carbonic anhydrase (CA) with aromatic heterocyclic sulfonamides was investigated in 2011 [12]. In another study, a thiabendazole sulfonamide showed a potent inhibitory activity against both mammalian and nematode α-CAs [13].

Five independently evolved classes of CAs (α, β, γ, δ, and ζ) have been identified, of which one or more are found in nearly every cell type, underscoring the general importance of this ubiquitous enzyme in nature [14]. The CAs are involved in several important biological processes, such as respiration and transportation of CO_2_ and bicarbonate between metabolizing tissues, pH and CO_2_ homeostasis, electrolyte secretion in different organs, bone resorption, calcification, tumorigenicity, and some biosynthetic reactions including gluconeogenesis, lipogenesis, and ureagenesis [15]. Since 1990, many demonstrated and putative β-CAs have been discovered not only in photosynthetic organisms, but also in eubacteria, yeast, archaeal species [16] and 18 metazoan species [17]. Recently, we reported 52 β-CAs in metazoan and protozoan species [18]. At least one study has shown the effects of β-CA inhibitors as anti-infective agents on different bacterial and fungal pathogens [19], yet this approach has not been tested *in vivo* in metazoans or protozoans. In this article, we introduce β-CAs as novel potential target enzymes to control agricultural and veterinary insects and parasites which cause enormous economic losses worldwide.

## Methods

### Identification of putative β-CA enzymes and multiple sequence alignment (MSA)

In total, 23 parasite and 8 plant β-CA sequences relevant to agriculture and livestock husbandry, or as model organisms, and one bacterial sequence (*Desulfosporosinus meridiei*) were retrieved from UniProt (http://www.uniprot.org/) and NCBI (http://www.ncbi.nlm.nih.gov/). The full list of agriculture and livestock husbandry pests and plants containing β-CA addressed in this research are shown in Table 1. We focused on 98 amino acid residues around the catalytic active site of all tested β-CAs, starting 7 amino acid residues prior to the first highly conserved sequence (CXDXR). The Clustal Omega algorithm [20] within the Jalview program (version 2.8.ob1) (http://www.jalview.org/) was used to create a multiple sequence alignment (MSA) [21].

**Table 1:** **Agriculture and livestock husbandry pests, and plants containing β-CA which applied in this research**

Species name	General name	Parasitic Features	Main concerns
***Acyrthosiphon pisum***	pea aphid	Sap-sucking in forage crops, such as peas, clover, alfalfa, and broad beans	Food canning industry [22]
***Ancylostoma caninum***	A species of phylum *Nematoda*	Infection of the small intestine of dogs and human (zoonosis)	Dog breeding [23]
***Ascaris suum*** **(** ***Ascaris lumbricoides*** **)**	large roundworm of pigs	Ascariasis in pig and human (zoonosis)	Pig breeding [24]
***Caligus clemensi***	Plural sea lice	Major ectoparasites of farmed and wild Atlantic salmon	Fishing and fish farming [25]
***Camponotus floridanus***	Carpenter ant	Nest in live or dead trees, rotting logs and stumps, buildings, telephone poles, and other wooden structures	Wooden instrument industries and consumers [26]
***Ceratitis capitata***	Mediterranean fruit fly (Medfly)	Causing extensive damage to a wide range of fruit crops	Invasion to orchards [27]
***Culex quinquefasciatus***	Southern house mosquito	Vector of West Nile virus (WNV), St. Louis encephalitis virus and other arboviruses, lymphatic filariasis, *Wuchereria bancrofti*, and *Plasmodium relictum* (avian malaria)	Zoonotic diseases which affect both humans and animals health [28]
***Dendroctonus ponderosae***	Mountain pine beetle (MPB)	Attacks to old or weakened trees, and speeds to younger forests	Wooden instrument industries and consumers [29]
***Entamoeba histolytica Entamoeba nuttalli Entamoeba dispar***	A genus of phylum *Amoebozoa*	Causative agent of amoebiasis in animals and human (zoonosis)	Humans and animals health [30]
***Haemonchus contortus***	Trichostrongyloid nematode (Red stomach worm, wire worm or barber’s pole worm)	Causative agent of Haemonchosis by blood feeding through attachment to abomasal mucosa of ruminants	Sheep and goat farming [31]
***Ichthyophthirius multifiliis***	Freshwater ich, or freshwater ick	White spot disease in freshwater fishes and rarely in human (zoonosis)	Fish and fish farming [32]
***Lepeophtheirus salmonis***	Salmon louse	Parasite living on wild salmon and fish farming	Fish and fish farming [25]
***Necator americanus***	New World hookworm	Necatoriasis in dog, cat, and human (zoonosis)	Humans and animals health [33]
***Solenopsis invicta***	Red imported fire ant (RIFA)	Mound-building activity, Damage plant roots which leads to loss of crops, and interfere with mechanical cultivation	Wooden instrument industries and consumers, and gardening [34]
***Tribolium castaneum***	Red flour beetle	Pest of stored grain products, carcinogenic by secretion of quinones, causative agent of occupational IgE-mediated allergy and some other diseases	Wheat, flour, cereal and nut based food industries [35–38]
***Trichinella spiralis***	Pork worm	Trichinosis in rat, pig, bear and human (zoonosis)	Pig breeding [39]
***Trichoplax adhaerens***	Adherent hairy plate	Adherence to the wall of a marine aquariums	Aquarium and ornamental fishing industry [40]
***Arabidopsis thaliana***	Mouse-ear cress	-	A popular model organism in plant biology and genetics [41]
***Pisum sativum***	Pea	-	Pea is most commonly the small spherical seed or the seed-pod [42]
***Gossypium hirsutum***	Upland cotton	-	Upland cotton is the most widely planted species of cotton [43]
***Nicotiana tabacum***	Tobacco	-	Its leaves are commercially processed into tobacco [44]
***Vitis vinifera***	Grape vine	-	Commercial significance for wine and table grape production [45]
***Solanum tuberosum***	Potato	-	The world’s fourth-largest food crop, following maize, wheat and rice [46]
***Populus trichocarpa***	Black cottonwood or California poplar	-	A model organism in plant biology [47]
***Capsella rubella***	A genus from Mustard family	-	A member of Mustard family [48]

### Phylogenetic analysis

All sequences were individually analyzed for completeness and quality. The β-CA sequence for *Solenopsis invicta* (UniProt ID: E9IP13) was determined to have a spurious exon when the genomic sequence was analyzed by the Exonerate program using the other β-CA proteins as query sequences, and subsequently 17 amino acids were removed [49]. Similarly, the full genome of *Acyrthosiphon pisum* was analyzed. Of the three *Acyrthosiphon pisum* β-CA sequences identified in UniProt, two were incomplete (UniProt IDs: C4WVD8 and J9JZY3) and found to be fragments of the same complete protein predicted in our analysis (*Acyrthosiphon pisum* BCA-2). Finally, the full genome of *Ichthyophthirius multifiliis* was scanned for β-CA proteins using the same method, and two new putative β-CA proteins were identified (*Ichthyophthirius multifiliis* BCA-3 and BCA-4).

A protein sequence alignment was created using Clustal Omega [20] based on which the corresponding nucleotide sequences were then codon-aligned by the Pal2Nal program [50]. Using the *Desulfosporosinus meridiei* bacterial sequence as an outgroup, a phylogenetic analysis was computed using Mr. Bayes v3.2 [51] with the GTR model of codon substitution and all other parameters set to default. In total, 200,000 generations were computed with a final standard deviation of split frequencies of 3.33 × 10^−4^. The final phylogenetic tree was visualized in FigTree (http://tree.bio.ed.ac.uk/software/figtree/).

### Prediction of subcellular localization

Subcellular localization of each identified invertebrate β-CA was predicted using the TargetP webserver (http://www.cbs.dtu.dk/services/TargetP/). TargetP is built from two layers of neural networks, where the first layer contains one dedicated network for each type of targeting sequences, such as cytoplasmic, mitochondrial, or secretory peptides, and the second layer is an integrating network that outputs the actual prediction (cTP = cytoplasmic, mTP = mitochondrial, SP = secretory, or other). It is able to discriminate between cTPs, mTPs, and SPs with sensitivities and specificities higher than what has been obtained with other available subcellular localization predictors [52].

### Prediction of antigenic sites in β-CA

The protein sequences of 23 parasite and 8 plant β-CAs were analyzed with the European Molecular Biology Open Software Suite (EMBOSS) program Antigenic (http://emboss.bioinformatics.nl/cgi-bin/emboss/antigenic). EMBOSS Antigenic predicts potentially antigenic regions of a protein sequence, using the method of Kolaskar and Tongaonkar [53]. Application of this method to a large number of proteins has shown that their accuracy is better than most of the known methods [54–56].

### Homology modelling

Homology models of four selected β-CAs, including FC551456 (*Ancylostoma caninum*), F1LE18 (*Ascaris suum*), E5SH53 (*Trichinella spiralis*), and C4LXK3 (*Entamoeba histolytica*) were prepared by first selecting the most suitable template structure. For this purpose, a BLAST search of the PDB database (http://www.rcsb.org/pdb/home/home.do) was performed using each of the four sequences. Results for three out of these four searches revealed that PDB structure 1EKJ (β-CA from *Pisum sativum*) possessed the most similar sequence, while PDB id 2A5V (β-CA from *Mycobacterium tuberculosis*) was found to be the most similar to C4LXK3 (*Entamoeba histolytica*). Clustal Omega was used to prepare a multiple sequence alignment for those six sequences.

The multiple sequence alignment showed nine completely conserved residues within the sequences; the known highly conserved CXDXR and HXXC motifs were among them (data not shown). Homology modelling was performed according to multiple sequence alignment containing FC551456 (*Ancylostoma caninum*), F1LE18 (*Ascaris suum*), E5SH53 (*Trichinella spiralis*), and PDB 1EKJ by using the Modeller program (version 9.13) [57] with PDB model 1EKJ (β-CA from *Pisum sativum*) as a template. A homology model for C4LXK3 (*Entamoeba histolytica*) was prepared using PDB 2A5V for pairwise alignment and as a template structure. The resulting models were structurally aligned using the BODIL program [58]. A figure illustrating the homology models was prepared by using the VMD program (version 1.9.1) [59], and edited within Adobe Photoshop (version 13.0.1).

The structural availability of the epitope in the PDB model 1EKJ (β-CA from *Pisum sativum*) and the homology model based on the β-CA sequence from *Ancylostoma caninum* was studied by preparing the molecular surface with VMD, using a probe radius of 1.4 Å. The potential epitope residues were excluded from the surface presentation and were shown as Van der Waals (VdW) spheres.

## Results

### Multiple sequence alignment (MSA)

**Figure 1:**
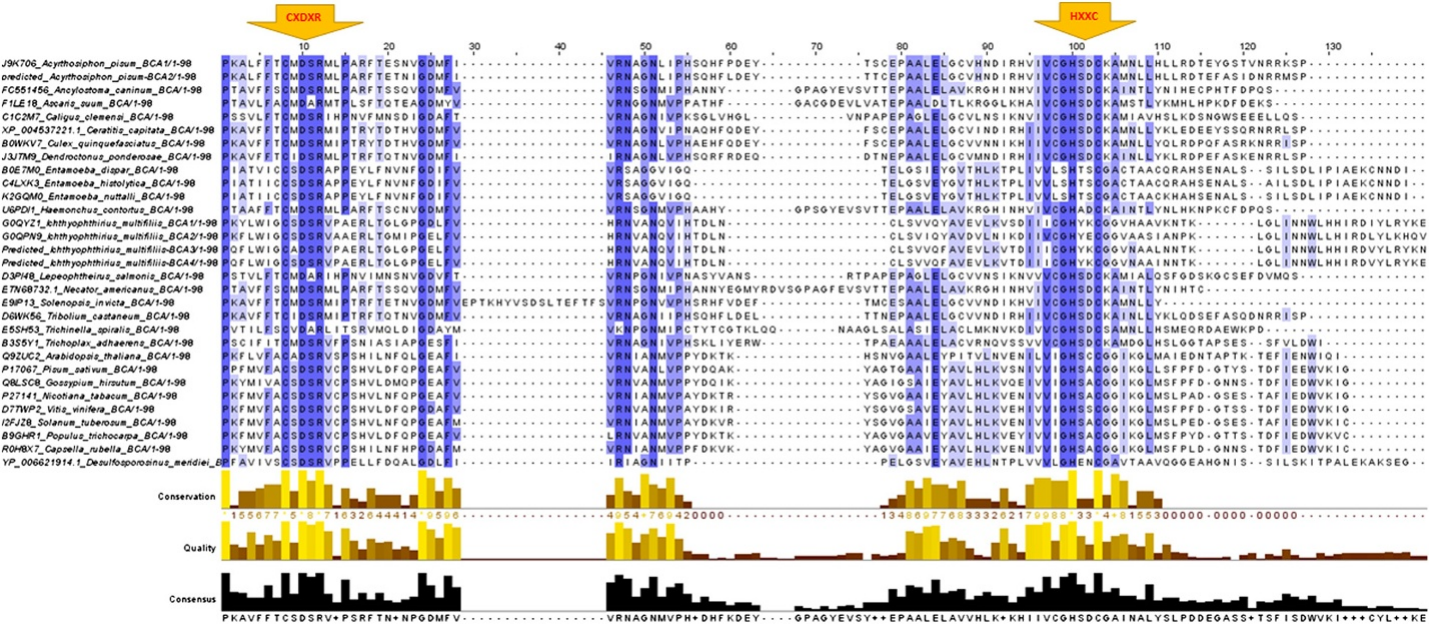
**Multiple sequence alignment of 23 parasite, 8 plant, and one bacterial (** ***Desulfosporosinus meridiei*** **) β-CA sequences showing the most conserved region of the active site.** The first (CXDXR) and second (HXXC) highly conserved sequences which are involved in zinc atom binding in catalytic active sites of the enzyme are marked with arrows at the top of the figure.

The MSA of 23 parasite and 8 plant β-CA sequences revealed the presence of the highly characteristic conserved sequence motifs CXDXR and HXXC (C: cysteine, D: aspartic acid, R: arginine, H: histidine, X: any residue) in all sequences. These results verify the presence of the β-CA enzyme in several insects and parasites which are pathogenic to various species of plants and animals and are thus considered relevant to agriculture and livestock husbandry (Figure 1).

### Phylogenetic analysis

**Figure 2:**
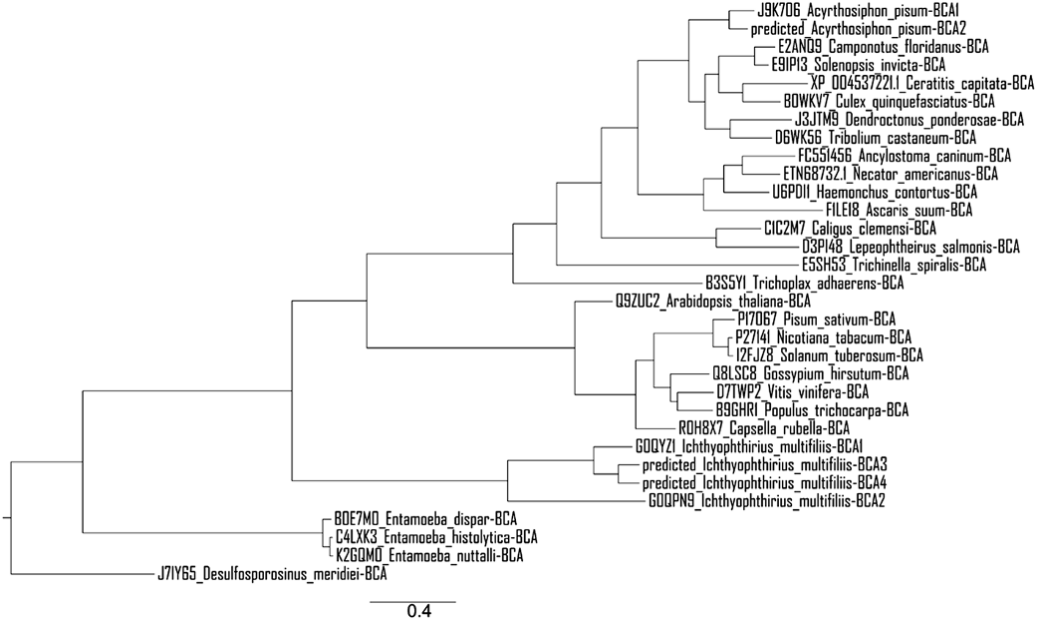
**Phylogenetic analysis of β-CAs from 23 parasite and 8 plant species.** β-CA from *Desulfosporosinus meridiei* was used as a bacterial outgroup.

The results of the phylogenetic analysis of DNA sequences encoding 23 parasite and 8 plant β-CAs are shown in Figure 2. From the resulting tree we see four distinct clades, three of which represent distinct potential β-CA targets. From the top, the first clade represents β-CAs of invertebrate pests, the second clade are plant model organisms, the third clade is entirely represented by the four β-CAs of *Ichthyophthirius multifiliis*, and the final clade represents three species of amoeba. The *Entamoeba spp*. sequences occupy a midpoint between our outgroup bacteria species and the others.

### Prediction of subcellular localization

The results of subcellular localization prediction of β-CAs in selected parasite and plant species are shown in Table 2. The predictions were based on the analysis of full-length β-CA protein sequences. In the *Name* column, there are both the UniProt ID and species scientific name. The results reveal that 6 of 23 β-CAs from parasites (XP_004537221.1, B0WKV7, U6PDI1, E5SH53, B3S5Y1, and predicted BCA2 in *A. pisum*) were predicted to have a mitochondrial localization signal; 6 of 8 β-CAs of plants (P17067, Q8LSC8, P27141, D7TWP2, I2FJZ8, and B9GHR1) were predicted to have a chloroplastic localization.

**Table 2:** **Prediction of subcellular localization of 23 pest and 8 plant β-CAs**

Species name	Entry ID	β-CA ID	cTP	mTP	SP	Other	RC	Loc
***Acyrthosiphon pisum***	J9K706	BCA1	-	0.473	0.050	0.631	5	-
***Acyrthosiphon pisum***	Predicted	BCA2	-	0.579	0.043	0.536	5	M
***Ancylostoma caninum***	FC551456	BCA	-	0.466	0.046	0.514	5	-
***Ascaris suum***	F1LE18	BCA	-	0.388	0.079	0.406	5	-
***Caligus clemensi***	C1C2M7	BCA	-	0.210	0.040	0.873	2	-
***Camponotus floridanus***	E2ANQ9	BCA	-	0.325	0.051	0.735	3	-
***Ceratitis capitata***	XP_004537221.1	BCA	-	0.549	0.039	0.512	5	M
***Culex quinquefasciatus***	B0WKV7	BCA	-	0.573	0.032	0.507	5	M
***Dendroctonus ponderosae***	J3JTM9	BCA	-	0.270	0.064	0.742	3	-
***Entamoeba dispar***	B0E7M0	BCA	-	0.114	0.158	0.766	2	-
***Entamoeba histolytica***	C4LXK3	BCA	-	0.113	0.151	0.779	2	-
***Entamoeba nuttalli***	K2GQM0	BCA	-	0.132	0.142	0.763	2	-
***Haemonchus contortus***	U6PDI1	BCA	-	0.587	0.057	0.403	5	M
***Ichthyophthirius multifiliis***	G0QYZ1	BCA1	-	0.071	0.046	0.946	1	-
***Ichthyophthirius multifiliis***	G0QPN9	BCA2	-	0.181	0.040	0.872	2	-
***Ichthyophthirius multifiliis***	Predicted	BCA3	-	0.059	0.078	0.954	1	-
***Ichthyophthirius multifiliis***	Predicted	BCA4	-	0.050	0.178	0.868	2	-
***Lepeophtheirus salmonis***	D3PI48	BCA	-	0.126	0.068	0.889	2	-
***Necator americanus***	ETN68732.1	BCA	-	0.379	0.036	0.604	4	-
***Solenopsis invicta***	E9IP13	BCA	-	0.326	0.052	0.756	3	-
***Tribolium castaneum***	D6WK56	BCA	-	0.054	0.097	0.938	1	-
***Trichinella spiralis***	E5SH53	BCA	-	0.876	0.028	0.177	2	M
***Trichoplax adhaerens***	B3S5Y1	BCA	-	0.582	0.038	0.459	5	M
***Arabidopsis thaliana***	Q9ZUC2	BCA	0.043	0.171	0.108	0.923	2	-
***Pisum sativum***	P17067	BCA	0.969	0.050	0.014	0.023	1	C
***Gossypium hirsutum***	Q8LSC8	BCA	0.947	0.154	0.008	0.019	2	C
***Nicotiana tabacum***	P27141	BCA	0.956	0.059	0.019	0.039	1	C
***Vitis vinifera***	D7TWP2	BCA	0.902	0.183	0.016	0.034	2	C
***Solanum tuberosum***	I2FJZ8	BCA	0.954	0.051	0.024	0.045	1	C
***Populus trichocarpa***	B9GHR1	BCA	0.931	0.231	0.021	0.012	2	C
***Capsella rubella***	R0H8X7	BCA	0.040	0.208	0.176	0.907	2	-

*cTP* = a chloroplast transit peptide, *mTP* = a mitochondrial targeting peptide, *SP* = secretory pathway, *Loc* (predicted localization) where *C* = chloroplastic, *M* = mitochondrial, *S* = secretory, − = other, *RC* = reliability class, from 1 to 5, where 1 indicates the strongest prediction. *RC* is a measure of the difference between the highest and the second highest output scores. There are 5 reliability classes, defined as follows: 1: diff ≥ 0.800, 2: 0.800 > diff ≥ 0.600, 3: 0.600 > diff ≥ 0.400, 4: 0.400 > diff ≥ 0.200 and 5: 0.200 > diff. Thus, the lower the value of RC the safer the prediction.

### Prediction of antigenic sites in β-CA

According to the acceptable 3–85 residue variation in epitope length of an antigen [60] and default parameters of EMBOSS Antigenic database, the minimum length of an antigenic region in this set of β-CAs is 6 amino acid residues. The predictions of antigenic sites in the 31 β-CA proteins are shown in Table 3; the highest score belongs to the most antigenic site.

**Table 3:** **Predicted antigenic sites of 23 pest and 8 plant β-CA primary sequences**

Species name	Entry ID	β-CA ID	Pest or plant	HitCount ^*^	The most antigenic epitope
***Acyrthosiphon pisum***	J9K706	BCA1	Pest	14	77 YTSCEPAALELGCVHNDIRHVIVCG***HSDC*** 105
***Acyrthosiphon pisum***	Predicted	BCA2	Pest	14	79 TCEPAALELGCVHNDIRHVIVCG***HSDC*** 105
***Ancylostoma caninum***	FC551456	BCA	Pest	11	101 INHVIVCG***HSDC***KAINTLYNIHECPHTFDP 130
***Ascaris suum***	F1LE18	BCA	Pest	15	102 KHAIVCG***HSDC***KAMST 117
***Caligus clemensi***	C1C2M7	BCA	Pest	10	84 EPAGLELGCVLNSIKNVIVCG***HSDC***KAMIAVHSL 117
***Camponotus floridanus***	E2ANQ9	BCA	Pest	11	80 CESAALELGCVVNDIRHVIVCG***HSDC*** 105
***Ceratitis capitata***	XP_004537221.1	BCA	Pest	13	72 HFQDEYFSCEPAALELGCVINDIRHIIVCGHSD 104
***Culex quinquefasciatus***	B0WKV7	BCA	Pest	14	75 DEYFSCEPAALELGCVVNNIKHIIVCG***HSDC*** 105
***Dendroctonus ponderosae***	J3JTM9	BCA	Pest	13	95 RHIIVCG***HSDC***KAINLLYKL 114
***Entamoeba dispar***	B0E7M0	BCA	Pest	8	85 SIEYGVTHLKTPLIVVLS***HTSC***GACTAACQRA 116
***Entamoeba histolytica***	C4LXK3	BCA	Pest	8	83 LGSVEYGVTHLKTPLIVVLS***HTSC***GACTAACQRA 116
***Entamoeba nuttalli***	K2GQM0	BCA	Pest	7	83 LGSVEYGVTHLKTPLIVVLS***HTSC***GACTAACKHA 116
***Haemonchus contortus***	U6PDI1	BCA	Pest	13	101 HINHVIVCGHADCKAINTLYNL 122
***Ichthyophthirius multifiliis***	G0QYZ1	BCA1	Pest	13	193 ANQVIHTDLNCLSVVQYAVEVLKVSDIIICG***HYKC***GGVHAAVKNT 237
***Ichthyophthirius multifiliis***	G0QPN9	BCA2	Pest	9	86 ANQVIHTDLNCLSVIQYAVDVLNIKDIIVCG***HYEC***GGVAASIANPKLGL 134
***Ichthyophthirius multifiliis***	Predicted	BCA3	Pest	7	65 ANQVIHTDLNCLSVVQFAVEVLKVTDIIICG***HYKC***GGVNAA 105
***Ichthyophthirius multifiliis***	Predicted	BCA4	Pest	6	62 ANQVIHTDLNCLSVVQFAVEVLKVTDIIICG***HYKC***GGVNA 101
***Lepeophtheirus salmonis***	D3PI48	BCA	Pest	10	82 PEPAGLELGCVVNSIKNVVVCG***HSDC***KAMIALQSF 116
***Necator americanus***	ETN68732.1	BCA	Pest	10	108 HINHVIVCGHSDCKAINTLYNIHTCPQ 134
***Solenopsis invicta***	E9IP13	BCA	Pest	14	97 CESAALELGCVVNDIKHVIVCG***HSDC*** 122
***Tribolium castaneum***	D6WK56	BCA	Pest	13	116 ALELGCVVNDIRHIIVCG***HSDC***KAINLLYKLQDS 149
***Trichinella spiralis***	E5SH53	BCA	Pest	11	100 KDIVVCG***HSDC*** 110
***Trichoplax adhaerens***	B3S5Y1	BCA	Pest	13	82 EAAALELACVRNQVSSVVVCG***HSDC*** 106
***Arabidopsis thaliana***	Q9ZUC2	BCA	Plant	13	80 PKFLVFA***CADSR***VSPSHILNFQ 101
***Pisum sativum***	P17067	BCA	Plant	16	153 PFMVFA***CSDSR***VCPSHVLDFQ 173
***Gossypium hirsutum***	Q8LSC8	BCA	Plant	14	151 KYMIVA***CSDSR***VCPSHVLDM 170
***Nicotiana tabacum***	P27141	BCA	Plant	15	146 KFMVFA***CSDSR***VCPSHVLNF 165
***Vitis vinifera***	D7TWP2	BCA	Plant	13	149 KFMVFA***CSDSR***VCPSHVLDFQ 169
***Solanum tuberosum***	I2FJZ8	BCA	Plant	15	146 KFMVFA***CSDSR***VCPSHVLNF 165
***Populus trichocarpa***	B9GHR1	BCA	Plant	13	146 KFMVFA***CSDSR***VCPSHVLDFQ 166
***Capsella rubella***	R0H8X7	BCA	Plant	11	84 KYMVFA***CSDSR***VCPSHILNFH 104

The italic and bolded residues represent the first (CXDXR) and second (HXXC) highly conserved sequences in the catalytic active sites of the enzyme whenever present in the predicted epitope.

*:HitCount means the total number of antigenic residues in the whole sequence of one protein or antigen.

### Homology modelling

**Figure 3:**
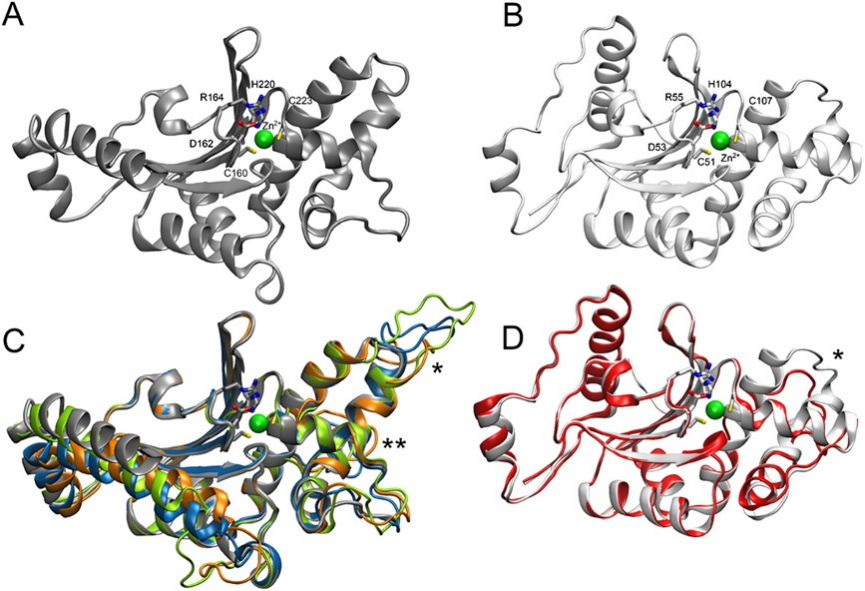
**Homology modelling. (A)** Cartoon presentation of β-CA from *Pisum sativum* (PDB 1EKJ). The Zn^2+^ion is shown as green sphere and the residues in direct contact with the ion are shown as liquorice models and labeled with residue numbers. **(B)** β-CA from *Mycobacterium tuberculosis* (PDB 2A5V, light gray). **(C)** Aligned homology models of β-CAs from *Ancylostoma caninum* (green), *Ascaris suum* (blue), and *Trichinella spiralis* (orange) are shown with PDB 1EKJ (gray). **(D)** Homology model of and *Entamoeba histolytica* (red) structurally aligned with PDB 2A5V (light gray). Highly variable loop regions are indicated by stars (“*” and “**”) **(C, D)**. Insertion suggested by homology models of *Ancylostoma caninum*, *Ascaris suum* and *Trichinella spiralis* is indicated by three stars **(C)**. The figure was prepared by using VMD (version 1.9.1).

Homology models of four selected β-CAs verified the predicted localization of conserved residues in the active site. Two loop regions showed high variability in the sequence length which is apparent in the Figure 3C, D and indicated by “*” and “**”. In addition, homology modelling suggested insertion located within the longest α-helix in case of homology models based on 1EKJ (Figure 3C, indicated by “***”).

**Figure 4:**
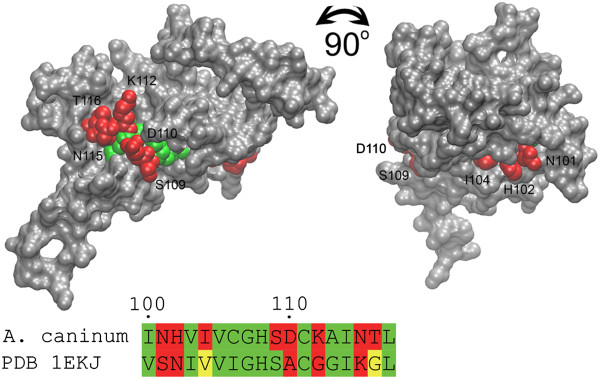
**Determination of the availability of the predicted epitope.** The molecular surface of the homology model of β-CA from *Ancylostoma caninum* is shown as solid grey and the target epitope sequence was excluded from the surface presentation. The epitope residues exposed to solvent are shown as red VdW spheres and numbered, while buried residues are shown with green spheres. An alignment containing PDB 1EKJ and the corresponding sequence from *Ancylostoma caninum* predicted β-CA is shown. The numbering of the residues in the alignment is according to the *Ancylostoma caninum* sequence. The yellow residues in the alignment indicate partially buried structure.

To study the molecular availability of the predicted main antigenic epitope, surface exposure of the homology model created from PDB model 1EKJ (β-CA from *Pisum sativum*) and the homology model based on the β-CA sequence from *Ancylostoma caninum* were studied by visualizing the molecular surface (Figure 4). The analysis revealed that the majority of the epitope was buried within the structure. The residues considered to be mainly buried in the structure are shown in green, while solvent-exposed residues are shown with red colour. Two residues in PDB model 1EKJ (β-CA from *Pisum sativum*) appear considerably smaller than their complements in the *Ancylostoma caninum*-based homology model, and those residues can be considered to be only partially exposed (Figure 4, indicated by yellow colour in the alignment). Taken together, these results indicate that the predicted epitope sequence is mainly buried in β-CA sequences.

## Discussion

Several insect, parasite, and plant genomes contain genes which encode β-CA enzymes. Some of these parasites and insects are either causative agents or vectors of important veterinary, fish farming, and zoonotic diseases (Table 1). For this analysis we selected 31 β-CAs, 23 from parasites and 8 from plants. These sequences were retrieved from protein databases, or predicted from their genomes, and all selected host or vector species are considered important for agriculture or livestock husbandry, or represent model organisms. The most significant species included *Ancylostoma caninum*, *Ascaris suum* (*Ascaris lumbricoides*), *Caligus clemensi*, *Culex quinquefasciatus*, *Entamoeba spp*, *Haemonchus contortus*, *Ichthyophthirius multifiliis*, *Lepeophtheirus salmonis*, *Necator americanus*, *Trichinella spiralis*, and *Trichoplax adhaerens*. One was an important pest in food industries (*Tribolium castaneum*). There was also an orchard invasive dipteran fruit fly (*Ceratitis capitata*) and three pests of wood industries, such as *Camponotus floridanus*, *Dendroctonus ponderosae*, and *Solenopsis invicta*.

Our MSA of β-CAs in plants, parasites, and insects showed that they all contain the first (CXDXR) and second (HXXC) highly conserved sequences of β-CA. The presence of β-CA proteins in various insects and parasites and their absence in mammals suggests that these enzymes could be potential targets for the development of novel pesticides or anti-parasitic drugs with minimal side effects on vertebrates. A key requirement for such novel β-CA inhibitors is the high isoform specificity. The distinction among β-CA proteins elucidated in the phylogenetic tree indicates that inhibitors can be created which would target β-CAs specific to different groups of species, leaving those in other species, such as plants, unaffected. Unfortunately, design of highly specific inhibitors will require proper structural data based on protein crystallography. Thus far, β-CA crystal structures from only a few different species are available in PDB database (http://www.rcsb.org/pdb/home/home.do), including some algae, bacteria, archaea, yeast, and a plant *Pisum sativum* [61].

**Figure 5:**
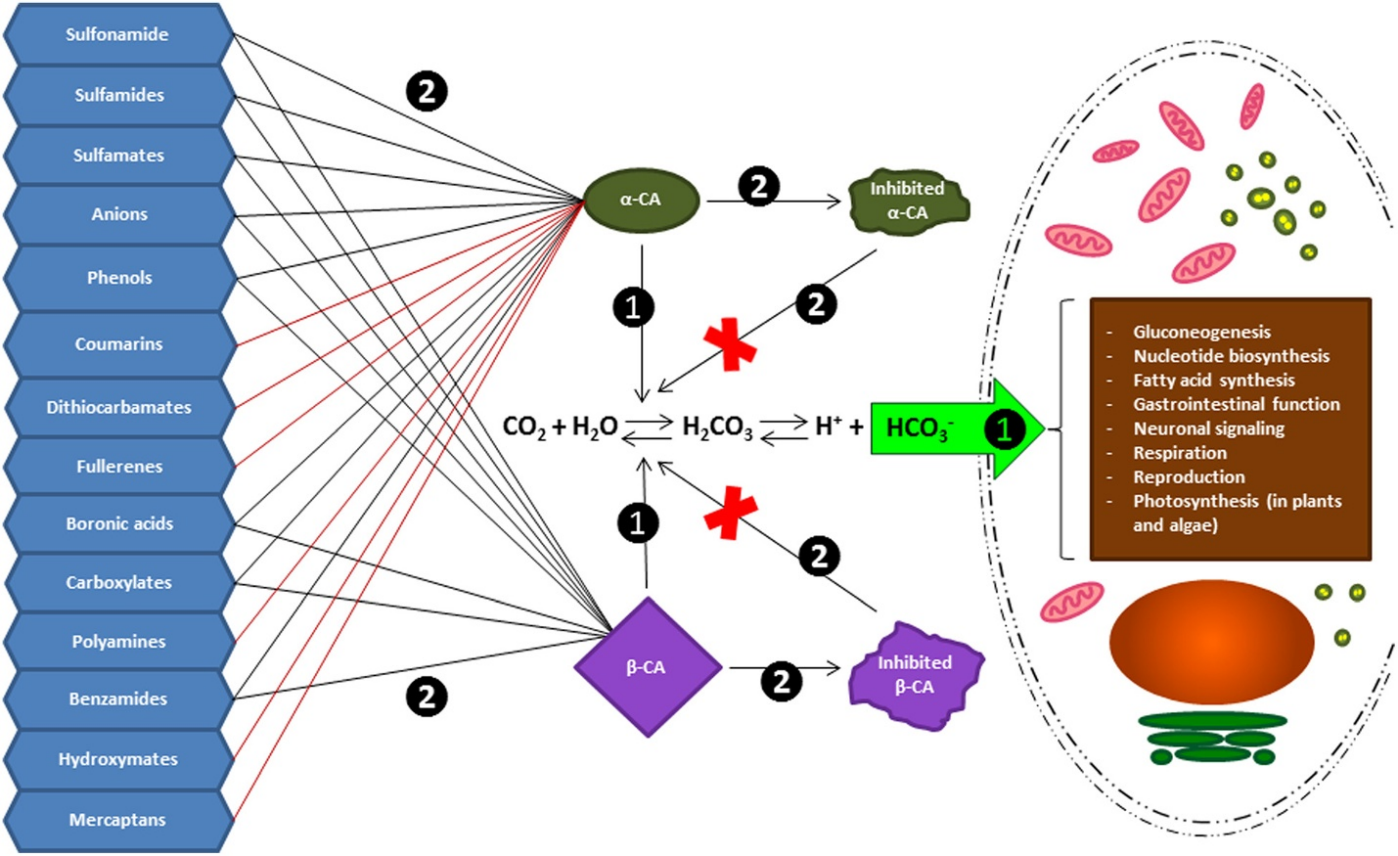
**Effects of 14 CA inhibitors on α**- **and β-CAs of parasites and insects. Some compounds inhibit members of both α- and β- CA enzyme families.** The brown box shows physiological processes where bicarbonate plays a role as a biochemical substrate. The ultimate goal of future research should be the creation of inhibitors specific to both enzyme families and to each isozyme. Ideally, the specific inhibitors would cause tissue- and organ-specific effects in parasites and vectors with minimal off-target effects on other species. Number 1 shows the catalytic pathway of α- and β-CA and number 2 shows the inhibitory effects of α- and β-CA inhibitors.

Our prediction results on the subcellular localization of β-CAs showed that 6 of 23 β-CAs from parasites (XP_004537221.1, B0WKV7, U6PDI1, E5SH53, B3S5Y1, and predicted BCA2 in *A. pisum*) are probably mitochondrial enzymes. It is well known that several pesticides have unwanted side effects because of their off-target impacts on mitochondria [62]. Blocking of β-CAs in insect and parasitic cells can affect mitochondrial metabolic cycles and possibly eradicate the pathogens. Figure 5 presents 14 categories of known α- and/or β-CA inhibitors, which are able to inhibit catalytic activity of these enzyme families [63, 64]. As the result, inhibition of CA activity would slow down some cellular biochemical pathways in parasites and insects, such as gluconeogenesis, nucleotide biosynthesis, fatty acid synthesis, gastrointestinal function, neuronal signaling, respiration, and reproduction. In plants and algae, it is known that β-CAs are required for CO_2_ sequestration within chloroplast, and therefore CA inhibition would affect the rate of photosynthesis [65]. Importantly, β-CA inhibition in fungi and *Drosophila melanogaster* revealed completely different inhibition profiles [17], suggesting that β-CAs of parasites and insects can be inhibited with higher affinity than plant CAs by applying the right inhibitors and concentrations.

Another important goal is to find inhibitors that are specific for β-CAs and do not affect α-CAs at all. This would first require detailed structural data on selected parasite and insect CAs. The resolved structures would then allow high throughput screening of chemical compounds, identification of the most promising inhibitor molecules, and testing of potential compounds *in vitro* and *in vivo*.

Vaccination would offer another option to develop anti-parasitic treatments based on β-CAs. In our study we used computational antigen prediction tools, which have been developed to reduce the laboratory work required to identify important antigenic epitopes in pathogenic proteins [66]. The Protegen database (http://www.violinet.org/protegen/) has been used to identify a number of predicted antigens from bacteria, viruses, parasites and fungi, which are involved in immune responses against various infectious and non-infectious diseases [67]. Antigenic site prediction of β-CA of parasites and plants revealed that the first and second highly conserved sequences (CXDXR and HXXC) represent the most plausible antigenic sites of β-CAs. Because these epitopes are located in the region of the active site and are mainly buried (Figure 4), they show very limited promise as vaccine targets. Furthermore, most β-CAs are intracellular proteins which are not readily accessible for immunological recognition. Taking all of these results together, small molecule inhibitors should still be considered the first option when β-CAs are investigated as therapeutic target proteins.

## Conclusions

Our present work is the first study that discusses the potential role of β-CAs as target proteins for pesticides and anti-parasitic agents in agriculture and livestock husbandry. Our results could potentially have significant impacts on development of novel pesticides, which would directly benefit both food and forest industries. This is important as pests cause significant costs for agricultural, horticultural, and livestock husbandry products due to production losses [68]. Since β-CA sequences are not present in the genomes of vertebrates, the possible off-target effects in human and vertebrate animals should be minimal if high isozyme specificity is achieved. Discovery and validation of a new generation of β-CA inhibitors as pesticides and anti-parasitic agents would be a novel research field for chemical and pharmaceutical industries to improve safe nutrition and general health in societies.
